# Correction: Long non-coding RNA MEG3 inhibits cell proliferation, migration, invasion and enhances apoptosis in non-small cell lung cancer cells by regulating the miR-31-5p/TIMP3 axis

**DOI:** 10.1039/d0ra90021d

**Published:** 2020-06-05

**Authors:** Kui Li, Xiaodan Wang, Zhen Huang, Hui Xu, Songbai Zheng, Yurong Qiu

**Affiliations:** Department of Translational Medicine Research Institute, Guangzhou Huayin Medical Laboratory Center. Ltd The Second Floor of Life Sciences Building of Southern Medical University No. 1838, North Guangzhou Street Guangzhou Guangdong China npivyd@163.com +86-18520035749; Technical Service Department, Guangzhou Huayin Medical Institute. Ltd Guangzhou Guangdong China

## Abstract

Correction for ‘Long non-coding RNA MEG3 inhibits cell proliferation, migration, invasion and enhances apoptosis in non-small cell lung cancer cells by regulating the miR-31-5p/TIMP3 axis’ by Kui Li *et al.*, *RSC Adv.*, 2019, **9**, 38200–38208, DOI: 10.1039/C9RA07880K.

In the published paper the Acknowledgements section was omitted; this should read:

This work was supported by Major Scientific and Technological Projects of Guangzhou Science and Technology Plan Projects (No. 201802020004).

The Royal Society of Chemistry apologises for these errors and any consequent inconvenience to authors and readers.

## Supplementary Material

